# Cell-Free DNA in Rheumatoid Arthritis

**DOI:** 10.3390/ijms22168941

**Published:** 2021-08-19

**Authors:** Teppei Hashimoto, Kohsuke Yoshida, Akira Hashiramoto, Kiyoshi Matsui

**Affiliations:** 1 Division of Diabetes, Endocrinology and Clinical Immunology, Department of Internal Medicine, Hyogo College of Medicine, Nishinomiya 6638501, Japan; k-matsui@hyo-med.ac.jp; 2 Department of Biophysics, Kobe University Graduate School of Health Sciences, Kobe 6540142, Japan; koh.yoshida1117@gmail.com (K.Y.); hash@med.kobe-u.ac.jp (A.H.)

**Keywords:** biomarker, cell-free DNA, rheumatoid arthritis, precision medicine

## Abstract

Endogenous DNA derived from the nuclei or mitochondria is released into the bloodstream following cell damage or death. Extracellular DNA, called cell-free DNA (cfDNA), is associated with various pathological conditions. Recently, multiple aspects of cfDNA have been assessed, including cfDNA levels, integrity, methylation, and mutations. Rheumatoid arthritis (RA) is the most common form of autoimmune arthritis, and treatment of RA has highly varied outcomes. cfDNA in patients with RA is elevated in peripheral blood and synovial fluid and is associated with disease activity. Profiling of cfDNA in patients with RA may then be utilized in various aspects of clinical practice, such as the prediction of prognosis and treatment responses; monitoring disease state; and as a diagnostic marker. In this review, we discuss cfDNA in patients with RA, particularly the sources of cfDNA and the correlation of cfDNA with RA pathogenesis. We also highlight the potential of analyzing cfDNA profiles to guide individualized treatment approaches for RA.

## 1. Introduction

Rheumatoid arthritis (RA) is a chronic inflammatory disease caused by genetic, epigenetic, and environmental factors [1]. Excessive expression of various inflammatory cytokines, such as interleukin (IL)-1β, tumor necrosis factor (TNF)-α, interferon (IFN)-γ, and IL-6, in synovial tissues plays an important role in disease progression [2,3]. Agents that target molecules involved in RA progression, such as disease-modifying antirheumatic drugs (DMARDs) and Janus kinase (JAK) inhibitors, have dramatically improved the management of RA [4]. Remission or low disease activity is an achievable goal for RA treatment; however, a subset of patients have partial or no response to treatment. The diversity of responses to currently available therapies suggests that RA is not a single disease entity [5]. Thus, new treatment strategies and markers for appropriate drug selection still need to be developed. Recent studies have associated various polymorphic genetic variants and epigenetic changes with the effectiveness of conventional synthetic DMARDs (csDMARDs) and biologic DMARDs (bDMARDs); thus, precision medicine based on the patient’s genetic background is expected to further improve RA management [6,7].

Fragmented cell-free DNA (cfDNA) is released into the bloodstream following damage or death of peripheral blood cells and tissue [8]. Serum levels of nuclear-derived cfDNA (cf-nDNA) are significantly higher in cancer patients than in healthy individuals, and serum cf-nDNA levels can be used to monitor the disease state [9,10,11]. Currently, various aspects of tumor-specific cell-free DNA (ctDNA), including serum ctDNA levels, the ratio between short and long fragments, DNA methylation, and gene mutations, can be monitored. Liquid biopsy is a non-invasive approach for detecting information about tumor-specific genetic and epigenetic alterations. This molecular profiling technique is gradually becoming the basis for “precision medicine” or individualized treatment [12,13]. Mitochondria-derived cfDNA (cf-mtDNA) has also been reported as a promising diagnostic and prognostic biomarker for several types of cancer [14,15].

In the field of rheumatic diseases, Tang et al. first reported in 1966 that serum cfDNA levels were elevated in patients with systemic lupus erythematosus (SLE) [16]. Since then, various studies have indicated the potential role of cfDNA in autoimmune diseases [17,18]. In patients with RA, cfDNA levels in peripheral blood and synovial fluid (SF) are elevated and have been associated with disease progression [19,20]. In addition, cfDNA induces joint inflammation via Toll-like receptor 9 (TLR9) pathways. Dong et al. reported that cfDNA in SF contains hypomethylated cytosine-phosphate-guanosine (CpG) motif-rich segments [21]. They also found that, in vitro, cfDNA from SF highly upregulated TNFα expression in TLR9-expressing THP-1 monocyte cell line, but not in TLR9-knockdown THP-1 cells [21]. Injection of the human TLR9 agonist CpG oligodeoxynucleotides (ODN) 2006 into the articular cavity of mice induced acute arthritis [22], and, similar to CpG-ODN 2006, an RA-associated CpG motif-rich sequence induced joint arthritis [21]. These results suggest the possibility of crosstalk between cfDNA and the innate immune system, and a correlation between cfDNA and the development of RA. In this review, we discuss the significance of cfDNA in the pathogenesis of RA. In particular, we focus on the potential of cfDNA as an RA biomarker, its involvement in inflammatory responses, and the possibility of individualized RA treatment based on cfDNA.

## 2. Circulating cfDNA

### 2.1. Mechanisms of cfDNA Release

cfDNA is typically released into circulating blood following cell death, such as apoptosis, necrosis, pyroptosis, ferroptosis, or extracellular trap-associated cell death (termed ETosis), and mainly consists of double-stranded nuclear DNA and mtDNA. Most cfDNA is packaged in the form of nucleosomes. In disease states, living cells also secrete large amounts of cfDNA, such as microparticles (MPs) or small membrane-bound vesicles [23,24]. Wang et al. reported that ctDNA released from cancer cells was not associated with apoptosis or necrosis but was proportional to the percentage of cells in the G1 phase, suggesting that ctDNA can also be released by living cells through exosomes and other organelles [25].

### 2.2. Apoptosis

Three major pathways of apoptosis have been recognized: extrinsic, intrinsic, and perforin/granzyme-mediated pathways, all of which lead to the activation of caspase-3, which triggers cell destruction [26,27]. Activated caspase-3 releases caspase-activated DNase (CAD), a DNA-specific endonuclease that cleaves double-stranded DNA in internucleosomal chromatin regions. Because CAD lacks exonuclease activity, it mainly breaks linker regions between nucleosomes [28], and most of the cfDNA derived from the nucleus is packaged in the form of nucleosomes or chromatosomes [29]. The size distribution of cfDNA has a dominant peak at around 167 base pairs (bp), which is similar to the length of DNA associated with a chromatosome. Additional peaks showed a 10.4-bp periodicity, which corresponds to the helical pitch of DNA in the nucleosome core (100–160 bp) [30].

### 2.3. Necrosis and Necroptosis

Necrosis, in contrast to apoptosis, is an accidental form of traumatic cell death that occurs as a result of acute cellular injury. It is characterized by cell membrane rupture due to unregulated mechanical or chemical events, leading to the release of intracellular contents, including cfDNA. Because of the non-specific digestion of chromatin, necrosis releases longer DNA fragments (>10 kbp) [31]. Necroptosis is another type of programmed cell death, but it presents with morphological features similar to those of necrosis. Necroptosis can be triggered by death receptors (DRs) (e.g., TNF superfamily receptors), IFN receptors, and TLR3/4 [32,33,34]. Activation of these receptors induces plasma membrane damage and the release of potential damage-associated molecular patterns (DAMPs), such as cfDNA [34].

### 2.4. Pyroptosis

Pyroptosis is a form of caspase-dependent and lytic cell death. However, pyroptosis differs from necrosis or necroptosis as it is a primary cellular response to stimuli following potentially damaging insults, such as pathogen ligands, DAMPs, altered levels of host metabolites, and environmental irritants [34]. Activated caspase-1 and caspase-4/5/11 cleave gasdermin D (GSDMD) and the released gasdermin-N domain binds to phosphoinositides in the plasma membrane and oligomerizes, resulting in cell swelling and eventual lysis. Finally, endogenous host molecules, including nDNA, mtDNA, and high mobility group box 1 protein (HMGB-1), are released into the extracellular space [35,36,37].

### 2.5. Ferroptosis

Ferroptosis is a type of programmed cell death that is induced by the accumulation of iron and reactive oxygen species (ROS) [38]. Its morphological features include mainly cytological changes, such as decreased or disrupted mitochondrial cristae, ruptured outer mitochondrial membrane, and severe mitochondrial membrane lipid peroxidation. However, the cell membrane remains intact, the nuclear size stays normal, and chromatin is not condensed. These cellular abnormalities result from the loss of selective permeability of the plasma membrane due to intense membrane lipid peroxidation and oxidative stress [39,40]. Although ferroptotic mechanisms are not yet fully elucidated, lipid peroxidation-mediated reduction in membrane fluidity or structural changes have been proposed to disrupt the function of the plasma membrane as a selective barrier, leading to excessive release of lipid oxidation products, such as proteins and DNA [41].

### 2.6. NETosis

Neutrophil extracellular traps (NETs) are networks of extracellular fibers composed of cytosolic and granule proteins. The antimicrobial components of NETs bind and kill bacteria, fungi, viruses, and parasites independent of phagocytic uptake. In addition, NETs can contribute to the pathogenesis of autoimmune diseases [42]. NET release results in neutrophil death through a pathway distinct from apoptosis or necrosis, referred to as neutrophil ETosis (NETosis). NETosis begins with nuclear delobulation and disassembly of the nuclear envelope. This process continues with the loss of cellular polarization, chromatin decondensation, and plasma membrane rupture, which releases histone-coated filamentous DNA into the extracellular space [43]. Majority of DNA in NETs/NETosis originates from the nucleus; however, NET structures also contain mtDNA [44]. ETosis occurs in various immune cells, including eosinophils, monocytes, mast cells, basophils, and macrophages, and result in the release of cfDNA [45,46].

## 3. cfDNA in RA

### 3.1. cfDNA in Peripheral Blood and Synovial Fluid

In the 1960s, Tan et al. and Barnett detected cfDNA in sera and SF from patients with RA [16,47]. In 1973, Koffller et al. investigated 54 RA patients [48] and showed that the concentration of serum cfDNA was elevated in RA patients compared with that in healthy controls (HC). Subsequently, several reports have shown elevated cfDNA levels in peripheral blood or SF [19,20,48,49]. In contrast, Dunaeva et al. observed reduced cfDNA levels in established RA cases compared to those in HC, and they did not detect any significant difference in serum cfDNA levels between HC and early RA cases [50]. A possible explanation for the differences in these results is that the different treatments reduced disease activity and may have inhibited the release of cfDNA into peripheral circulation.

### 3.2. Association between cfDNA and Disease Activity

Leon et al. found that plasma cfDNA correlated with symptom severity and the extent of tissue damage [19]. In recent studies, cfDNA levels in patients with RA correlated with parameters of disease activity, such as C-reactive protein (CRP) levels, erythrocyte sedimentation rate (ESR), and disease activity score (DAS28) [51,52,53]. Rykova et al. also reported that circulating nDNA and DAS28 had a tendency towards correlation, but they found no association between CRP levels and cell surface-bound (csb)-nDNA or csb-mtDNA [54]. In addition, cfDNA levels in the SF are correlated with CRP [55]. In contrast to the results of these studies, cf-mtDNA in plasma or in SF did not correlate with disease activity [56]. Notably, serum fetal cfDNA levels in pregnant women with well-controlled RA were higher than in those with high disease activity [57]. Thus, most studies have reported an association with disease activity, although the correlation is not strong (see summary in Table 1).

### 3.3. Response to Treatment

Rykova et al. examined the concentrations of circulating nDNA and mtDNA in RA patients as potential biomarkers for response to bDMARDs (MTX/etoricoxib or rituximab/MTX). There was no difference in cf-nDNA, cf-mtDNA, csb-nDNA, and csb-mtDNA levels between the two treatment groups [54]. In our study, we measured the concentration of cfDNA in plasma every 4 weeks starting from baseline to 24 weeks in 30 patients treated with bDMARDs (*n* = 10 treated with a TNF inhibitor; *n* = 8 with tocilizumab, and *n* = 12 with abatacept). We found that cfDNA levels in good responders were elevated at weeks 4 or 8, while those in moderate or non-responders were not. The temporary elevation in cfDNA levels in peripheral blood is assumed to be due to the absorption of SF or due to apoptosis of synovial cells, and our results indicate the potential of using cfDNA for predicting the early therapeutic effects of bDMARDs [49].

In contrast, Laukova et al. evaluated cfDNA and disease activity in 32 patients with RA after initiating bDMARD treatment at baseline, week 12 and week 24 (the types of bDMARDs were not indicated). Their results showed that both DAS28 and CRP levels decreased after 3 months of treatment, and plasma cf-nDNA and cf-mtDNA levels decreased after 6 months. Therefore, they concluded that cfDNA was not a useful biomarker for disease activity [52]. Because these studies differ in cfDNA evaluation points, patient backgrounds, and therapeutic agents, the reported outcomes cannot be directly compared. In fact, when cfDNA levels were measured before and 1 h after infliximab infusion, marked changes in cfDNA levels were observed in 7 out of 10 RA patients [58]. Considering these results, cfDNA levels after treatment may be influenced by several factors, including the timing of blood withdrawal and the type of bDMARDs. Therefore, in order to understand the association between cfDNA and therapeutic response to each bDMARD, it is necessary to evaluate large sample sizes with varying bDMARD treatments over both short and long durations.

## 4. Origins of cfDNA in RA

### 4.1. Neutrophils

Various types of cell death are essential for RA progression; however, the origin of cfDNA in RA remains unclear. cfDNA levels in SF of patients with RA were significantly higher than those in peripheral blood of patients with RA and those in SF of patients with osteoarthritis (OA) [49]. Moreover, proteins from neutrophils strongly correlated with cfDNA levels in SF from patients with RA, indicating that cfDNA originated from neutrophils. Although apoptosis of neutrophils is delayed in RA [59,60], inflammatory cytokines, fibroblast-like synoviocytes (FLS), and B cells are potential stimuli for enhancing NETs/NETosis. In a recent study, Birkelund et al. performed proteomic analysis of SF from patients with RA and with spondyloarthritis (SpA), and found that large amounts of inflammatory proteins, including neutrophil-derived proteins, were detected in SF from RA cases, but not in SF from SpA cases. These results suggest that NETs/NETosis in the RA synovium produces cfDNA in the RA-SF [55].

### 4.2. Macrophages, Monocytes and FLS

**Figure 1:**
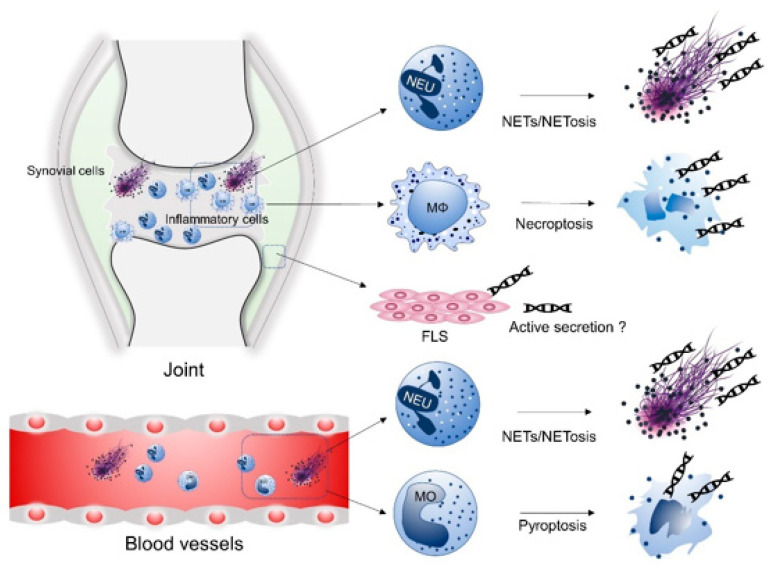
The origin of cell-free DNA (cfDNA) in rheumatoid arthritis (RA). Various cells, such as neutrophils, macrophages, monocytes, and fibroblast-like synoviocytes (FLS), are the hypothesized origins of cfDNA. In the synovial fluid, neutrophil extracellular traps (NETs), or NET cell death (NETosis), produce cfDNA. Tumor necrosis factor alpha (TNFα)-induced necroptosis in FLS-macrophages and macrophages also releases cfDNA. Tumor-like rheumatoid FLS are also likely sources of cfDNA. In peripheral blood, neutrophils provide cfDNA through NETs/NETosis, and monocytes may also release cfDNA following pyroptosis.

In addition to neutrophils, various cell types, including macrophages, monocytes, and FLS, are believed to release cfDNA in RA (Figure 1). Treatment with TNF-α induced necroptosis in macrophages from synovial tissue from patients with RA but not from patients with OA [61]. Furthermore, serum from RA patients induced GSDMD-dependent pyroptosis in monocytes, which was associated with disease activity and the elevation of cfDNA levels in SF and peripheral blood [62]. In addition, both the activation of proto-oncogenes and the inactivation of tumor suppressor genes in rheumatoid FLS led to tumor-like characteristics of FLS, indicating a molecular basis for pannus formation. Similar to cancer cells, tumor-like rheumatoid FLS may also be a source of cfDNA [63,64,65,66].

Methods for identifying the origin of cfDNA have been reported by two groups. Nucleosome positioning varies among cell types. Examination of the nucleosome positioning profiles of cfDNA indicated the origin of cfDNA [30]. The other group reported that each cell type has a unique DNA methylation pattern, and they described the method for investigating cfDNA in specific marker loci using next-generation sequencing (NGS) or droplet digital polymerase chain reaction [67,68].

It is important to confirm the source of cfDNA in RA to understand how cfDNA may be utilized for RA diagnosis, prognosis, or monitoring. Notably, the DNA methylation pattern in SF has been reported to be similar to that in synovial cells [69], suggesting the presence of synovial cell-derived cfDNA in SF. Apoptosis is suppressed in synovial cells [70]; however, treatment with DMARDs and bDMARDs induced apoptosis of RA-FLS. In fact, Nakazawa et al. reported that MTX induced apoptosis in FLS both in vivo and in vitro [71]. Recently, we have clarified the mechanism of MTX-induced apoptosis. MTX upregulates the circadian transcriptional factor proline- and acidic amino acid-rich basic leucine zipper, resulting in RA-FLS apoptosis [72]. TNF inhibitors also induce apoptosis in RA-FLS [73,74]. Thus, cfDNA may be released after FLS apoptosis as a result of treatment. However, further studies will have to be performed to determine the origin of cfDNA in patients with RA.

## 5. cfDNA-Induced Inflammation

### 5.1. Recognition of Self-DNA

**Figure 2:**
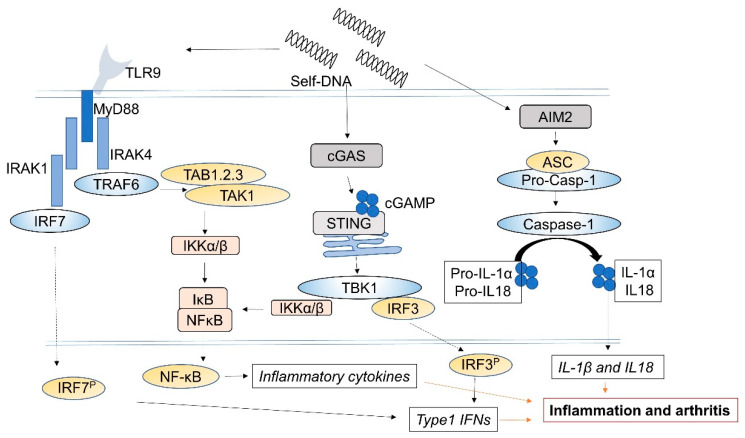
Recognition of self-DNA by DNA sensing receptors. Toll-like receptor 9 (TLR9) recognizes CpG motifs in cell-free DNA (cfDNA) and activates the transcription factor nuclear factor (NF)-κB and interferon regulatory factor (IRF)-7 through the myeloid differentiation primary response gene 88 (MyD88)-dependent pathway. cGAS, a cytosolic DNA sensor, catalyzes the synthesis of cyclic GMP-AMP (cGAMP) from ATP and GTP. 2′3-cGAMP binds to the endoplasmic reticulum (ER) adaptor, stimulator of interferon genes (STING). STING then activates kinase TANK-binding kinase 1 (TBK1) and IκB kinase (IKK) that phosphorylate the transcription factor IRF3 to turn on the expression of NF-κB and other in Figure 1 and IL-6. The absent in melanoma-2 (AIM2) inflammasome recognizes self-DNA and activates apoptosis-associated speck-like protein containing CARD (ASC), resulting in the secretion of IL-1β and IL-18.

Various DNA sensors such as TLR9, cyclic GMP-AMP synthase (cGAS), and absent in melanoma-2 (AIM-2)-like receptors have been recognized as potent DAMPs in the host cell. Host DNA in the nucleus and mitochondria allows the maintenance of self-tolerance; however, under stress conditions, host DNA enters the cytosol following nuclear or mitochondrial damage [75]. The recognition of cfDNA as a DAMP leads to the development of arthritis or inflammatory disease and is associated with the pathogenesis of RA (Figure 2).

### 5.2. TLR9 and cGAS

TLR9 is a receptor for DNA sequences that contain unmethylated CpG motifs. It activates the transcription factor nuclear factor (NF)-κB and interferon regulatory factor (IRF)-7 through myeloid differentiation primary response gene 88 (MyD88)-dependent pathway [76]. Macrophages in the joints produce TNF-α, which induces arthritis in response to bacterial DNA or mtDNA [77,78]. Immune complexes containing self-DNA activate RA-specific B cells [79]. In these cases, TLR9 is important for activating the *TNFα* gene in macrophages. In contrast, Kawane et al. reported that DNase 2 knockout mice developed arthritis via a TLR-independent mechanism [80].

cGAS, a cytosolic DNA sensor, catalyzes the synthesis of cyclic GMP-AMP (cGAMP) from ATP and GTP. 2′3-cGAMP binds the endoplasmic reticulum (ER) adaptor stimulator of interferon genes (STING). STING then activates TANK-binding kinase 1 (TBK1) and IκB kinase (IKK), which phosphorylate the transcription factor IRF3 to turn on the expression of NF-κB and other inflammatory cytokines such as type I interferons, TNF, IL-1β, and IL-6 [81]. Under TNF-α stimulation, cGAS was overexpressed in RA-FLS and enhanced the production of inflammatory cytokines and Akt, promoted the phosphorylation of extracellular signal-regulated protein kinase (ERK) [82]. Furthermore, the accumulation of cytosolic DNA promoted an inflammatory response via the cGAS-STING pathway in RA FLS [83].

Recently, Liang et al. demonstrated that intravenous injection of cationic nanoparticles reduced arthritic symptoms, including tissue swelling and bone and cartilage damage, in a CpG-induced mouse model and in a collagen-induced arthritis rat model [22]. These studies suggest the possibility of new treatments that target DNA sensors, including TLR-9 and cGAS. Moreover, SF-cfDNA from RA patients, which is enriched with hypomethylated CpG motif-rich sequences, induced severe inflammatory responses in vitro [21]. They demonstrated, for the first time, that SF-cfDNA is important for the development of RA.

### 5.3. AIM2

The AIM2 inflammasome recognizes dsDNA from viruses, bacteria, and mtDNA, and allows complex oligomerization, and its interaction with the adaptor protein, apoptosis-associated speck-like protein containing CARD (ASC) results in the secretion of IL-1β and IL-18 [84]. AIM2 has also been reported to recognize cytosolic self-DNA (80–300 bp), leading to the development of psoriasis, arthritis, and other autoimmune diseases [85]. Indeed, AIM2 was shown to be upregulated in RA but not in OA synovium [86]. CD14 co-expressing AIM2 cells did not significantly differ between RA patients and healthy subjects. However, the AIM2 expression levels were lower in the group of patients with DAS28 > 4 than in the control group, especially in late-stage disease (>2 years). These results showed that monocytes from patients with RA were prone to release IL-1β in the absence of the AIM2 inflammasome signal [87]. Chen et al. also reported that AIM2 expression in RA sera was lower than that in HC sera. In contrast, AIM2 was expressed in the cytoplasm of FLS, and AIM2 protein and mRNA levels were relatively higher in FLS from patients with RA than in patients with OA. The H scores, a semiquantitative immunohistochemical parameter [88], for AIM2 and IL-1β correlated with ESR and CRP levels in patients with RA. Moreover, RNA silencing of AIM2 expression inhibited FLS proliferation [89]. Thus, the AIM2 inflammasome pathway in FLS may be involved in the pathogenesis of RA and may be a therapeutic target.

## 6. Liquid Biopsy and Precision Medicine

### 6.1. Liquid Biopsy in Cancer

Non-invasive liquid biopsy was introduced in the oncology field in 2010s. This method is gradually replacing tissue diagnosis as the method for obtaining important information for predicting prognosis, therapeutic responses, and relapse in cancer [90,91]. Genomic alterations in ctDNA provide more accurate information than the analysis of tumor tissues for monitoring disease states and predicting prognosis. In particular, PCR-based ctDNA assays for epidermal growth factor receptor (EGFR) mutations and Ras have been used to determine treatment plans [92]. Furthermore, PCR-based analysis is a sensitive and inexpensive approach for screening for well-known genetic variants.

NGS is capable of detecting mutant allele fractions with low frequency (<1%), and a number of approaches, including the use of unique molecular identifiers or unique barcodes, can help increase the sensitivity and reduce false negative results of NGS analyses [93]. The rapid development of NGS technologies can achieve higher sensitivity than tissue biopsy and can be designed for different purposes, such as diagnosis and treatment [94]. However, the trade-off between sensitivity and cost remains the greatest concern for clinical practice [93].

### 6.2. Genetic Variants in RA

In patients with RA, personalized medicine based on cfDNA has not yet been applied. However, genome-wide association studies (GWAS) have significantly increased the amount of genetic information obtained from patients with RA [95,96,97,98], revealing various genes that are linked to RA pathogenesis. Among these, polymorphic genetic variants are particularly important for the development of personalized medicine [99] and have been found to affect responses to treatment, such as csDMARDs, bDMARDs, and targeted synthesis DMARDs [7]. Polymorphisms in certain genes, including *AMPD1 34C > T* and *ATIC T675C,* predict responsiveness to MTX in patients with RA [100]. The *MTHFR C677T* polymorphism was associated with MTX toxicity [101], and the *ATIC 347 C/G* polymorphism correlated with non-responsiveness to or toxic responses to MTX in Caucasian patients with RA [102]. Various polymorphic variants of the genes, such as *TNF, TNFA, TNFR1A36A, TLR1, TLR5, MED15* and *MAFB*, have also been associated with response to TNF inhibitors [103,104,105,106]. The efficacy of tocilizumab was associated with an *IL6R* polymorphism [107]. Quartuccio et al. reported that the V allele (158 V/F) of *FCGR3A* increased response to rituximab in patients with RA. Patients bearing this allele were less likely to lose response to the drug after 4–6 months of treatment [108], suggesting that the gene is useful for determining the efficacy of rituximab treatment [109].

### 6.3. DNA Methylation in RA

In contrast to genetic mutations, epigenetic changes are reversible. Epigenetic modifications also play an important role in gene expression [110]. The most commonly studied epigenetic modification is DNA methylation, wherein DNA methyltransferases (DNMTs) add −CH_3_ group to the carbon 5 position of cytosine [111]. It is an essential epigenetic modification that determines cell survival through its effects on gene expression regulation, retrotransposon inactivation, and X-chromosome inactivation. Richardson et al. first reported hypomethylation of genomic DNA in T cells and mononuclear cells in RA [112]. The expression of 5-methylcytocine (5mC) in synovial tissue has also been reported to be lower in RA than in OA [113]. Interestingly, genome-wide DNA methylation analysis revealed that RA synovial cell-derived FLS have disease-specific methylation patterns, and DNA methylation levels are different in a number of loci involved in RA pathology [114,115,116]. Furthermore, Nakano et al. demonstrated that TNFα and IL-1β suppress the expression of DNMTs in FLS and induce demethylation of the entire genome [117].

To identify new therapeutic targets, Whitaker et al. combined GWAS with DNA methylation analysis to determine genetic and epigenetic differences between RA SF and OA SF. A number of genes, such as *ELMO1*, *LBH*, and *PTPN11*, were found to be directly involved in RA pathogenesis and can be used as therapeutic targets [118]. Thus, DNA methylation can be used to determine future treatment targets and responses [119]. In addition, differential DNA methylation patterns for the *LRPAP1* gene have been shown to be a potential biomarker of response to TNF inhibitors [120]. Higher baseline global leukocyte DNA methylation was shown to be associated with less decrease in DAS28 and non-response to MTX [121]. Thus, DNA methylation affects responses to treatment, and methylation analysis of cfDNA may be helpful for the development of precision medicine for RA.

## 7. Prospective

Whether cfDNA is a useful predictor of response to treatments is the most important question that needs to be answered. Large multicenter, prospective, observational studies are needed to convincingly answer this question, because cfDNA levels in peripheral blood vary according to the type of treatment, or according to the presence of advanced synovitis. The most appropriate strategy would be to compare each treatment group, including TNF-inhibitors, IL-6 inhibitors, abatacept, rituximab and JAK inhibitors, with MTX-treated. If it is possible to assess the amount of short-term changes in cfDNA levels in the first few weeks of treatment, synovial apoptosis in response to treatment can be detected at a very early stage.

The next important question is whether cfDNA itself is a therapeutic target. cfDNA is recognized as DAMP by DNA sensors, such as TLR9 and cGAS. Thus, scavengers of DAMP molecules offer a new modality of treating inflammation [122]. Indeed, the removal of DAMP molecules in vivo using nucleic acid-binding polymers has been reported to improve a variety of conditions including arthritis [22], cancer [123,124] and sepsis [125,126].

The final issue that needs to be considered is whether cfDNA can be used for precision medicine. The advantage of cfDNA is that it can be easily extracted from peripheral blood or joint fluid. Therefore, using cfDNA to analyze genetic polymorphisms in *TNFA* and *TNFR1A36A*, we can find out whether the cells respond to TNF-I being used. In the future, gene analysis of cfDNA could be used to develop gene therapy for RA. For example, the recently reported RA-associated gene *ELMO1* is one of the potential targets for gene therapy [118,127]. cfDNA analysis of mutations and methylation changes in *ELMO1* may help determine whether inhibition of this gene is useful. This treatment strategy is feasible like the one using EGFR tyrosine kinase for lung cancer with mutations in EGFR. Therefore, many promising therapeutic target genes will be discovered in RA, and gene retrieval using cfDNA may lead to new breakthroughs in the treatment of RA.

## 8. Conclusions

We have discussed how cfDNA can serve as a biomarker for RA progression, treatment responses, and prognosis based on cfDNA involvement in inflammatory pathogenesis and its correlation with several genetic indicators of disease. The analysis of extracellular DNA is an interesting area of study for RA pathogenesis and treatment. In particular, precision medicine, which is currently becoming the mainstream treatment for malignancies, is a promising therapeutic strategy for RA. Research on cfDNA and its potential as a biomarker for disease progression and treatment responses is continuing to grow and is expected to contribute to the field of personalized medicine.

## Figures and Tables

**Table 1:** Association between cfDNA and disease activity.

Author, Year, Reference	Source, Number of Patients (*n*)	Target	Results
Leon et al., 1977 [19]	Serum, *n* = 72	nDNA	High levels of cfDNA were found in patients with severe symptoms
Leon et al., 1981 [20]	Serum, *n* = 47	nDNA	Positive association with disease activity (leukocytes, protein levels in SF, and ESR)
	SF, *n* = 47		
Hajizadeh et al., 2003 [56]	Plasma, *n* = 54	mtDNA	No significant association with CRP, erosivity or extra-articular manifestations
	SF, *n* = 54		
Zhen et al., 2006 [57]	Serum, *n* = 25	Fetal DNA	Fetal DNA levels were significantly higher in pregnant women with well-controlled disease than in those with active disease
Abdelal et al., 2016 [51]	Plasma, *n* = 30	nDNA	Positive association with ESR (*r* = 0.38, *p* = 0.04)
			Positive association with CRP (*r* = 0.34, *p* = 0.04)
			Positive association with DAS28 (*r* = 0.52, *p* = 0.005)
Rykova et al., 2017 [54]	Plasma, *n* = 74	nDNA	Tendency for association with DAS28 (*r* = 0.31, *p* = 0.06)
			nDNA levels were higher in high disease activity group than in low and moderate disease group
		csb nDNA	No association with CRP (*r* = −0.095, *p* = 0.46)
		mtDNA	Tendency for association with CRP (*r* = 0.24, *p* = 0.06)
		csb mtDNA	No association with CRP (*r* = 0.04, *p* = 0.76)
Laukova et al., 2018 [52]	plasma, *n* = 32	ecDNA	Positive association with ESR (*r* = 0.33, *p* < 0.01)
			Positive association with CRP (*r* = 0.25, *p* < 0.05)
			Positive association with DAS28 (*r* = 0.27, *p* < 0.01)
Eldosoky et al., 2018 [53]	Plasma, *n* = 35	nDNA	Higher in the active group than in the remission group
			Positive association with ESR (*r* = 0.78, *p* < 0.001)
			Positive association with DAS28 and ESR (*r* = 0.85, *p* < 0.001)
Bikelund et al., 2020 [55]	SF, *n* = 32	nDNA	Positive association with CRP (*r* = 0.48, *p* < 0.001)

SF: synovial fluid; cfDNA: cell-free DNA; nDNA: nuclear DNA; mtDNA: mitochondrial DNA; csbDNA: cell surface-bound DNA; ecDNA: extracellular DNA; ESR: erythrocyte sedimentation rate; CRP: C reactive protein; DAS28: disease activity score.

## Data Availability

Not applicable.
